# What is the impact of integrated care on the job satisfaction of primary healthcare providers: a systematic review

**DOI:** 10.1186/s12960-023-00874-w

**Published:** 2023-11-01

**Authors:** Mei Liu, Jian Wang, Jiaxu Lou, Ruonan Zhao, Jiahui Deng, Ziyu Liu

**Affiliations:** 1 Centre for Health Management and Policy Research, School of Public Health, Cheeloo College of Medicine, Shandong University, Jinan, 250012 China; 2 NHC Key Lab of Health Economics and Policy Research, Shandong University, Jinan, 250012 China

**Keywords:** Primary care providers, Integrated care, Job satisfaction

## Abstract

**Background and objectives:**

The integration of care influenced the job satisfaction of healthcare professionals, especially affecting primary healthcare providers (PCPs). This study aimed to perform a systematic review to explore the impact of integrated care on the job satisfaction of PCPs on the basis of Herzberg’s two-factor theory.

**Methods:**

This review was conducted following the Preferred Reporting Items for Systematic Reviews and Meta-Analyses (PRISMA) guidelines. We searched 6 electronic databases, including CNKI, WANFANG, PubMed, Web of Science, Cochrane Library, and Embase. Data were retrieved from inception to 19 March 2023. The Mixed Methods Appraisal Tool (MMAT) version 2018 was used to assess the methodological quality of studies for inclusion in the review.

**Results:**

A total of 805 articles were retrieved from databases, of which 29 were included in this review. 2 categories, 9 themes, and 14 sub-themes were derived from the data. 2 categories were identified as intrinsic and extrinsic factors. Intrinsic factors included 4 themes: responsibilities, promotion opportunities, recognition, and a sense of personal achievements and growth. Extrinsic factors included 5 themes: salaries and benefits, organizational policy and administration, interpersonal relationships, working conditions, and work status. To specify some key information under certain themes, we also identify sub-themes, such as the sub-theme “workload”, “work stress”, and “burnout” under the theme “work status”.

**Conclusions:**

Findings suggested that the integration of care had both negative and positive effects on the job satisfaction of PCPs and the effects were different depending on the types of integration. Since PCPs played a vital role in the successful integration of care, their job satisfaction was an important issue that should be carefully considered when implementing the integration of care.

## Background

The aging population and the growing prevalence of chronic diseases posed a great challenge to healthcare systems worldwide, especially in countries, where healthcare was fragmented [1–3]. To improve the quality and accessibility of healthcare while reducing the general cost, many countries initiated reforms to promote the integration of care, such as the Kaiser Healthcare Group of the United States and the *Yilianti*^1^ of China [5–8].

Primary healthcare providers (PCPs) played an essential role in promoting the integration of care, especially concerning the continuity and coordination of healthcare [9–13]. The job satisfaction of PCPs is recognized as an essential pillar of healthcare systems worldwide, which is known to be associated with several factors [14]. Many factors have been well-explored by the literature including systematic reviews [14–22]. Along with the integrated care reform, there was a growing evidence base for the correlation between the integration of care and the job satisfaction of PCPs. On one hand, studies reported that the job satisfaction of PCPs affected the quality and coordination of healthcare by influencing their job performance, job stability, and teamwork [23–27]. On the other hand, a growing literature explored the influence of integrated care on the job satisfaction of PCPs but the findings were inconsistent across studies. Some studies found that the integration of care enhanced the job satisfaction of PCPs by improving their working conditions, career development, and interpersonal relationships [11, 28–32]. Whereas, some studies claimed that integrated care generated a negative influence on the job satisfaction of PCPs because of the extra workload and job stress [30, 33, 34]. Due to the inconsistency of findings across studies, little seems known about how the integration of care impacts the job satisfaction of PCPs. Identifying job satisfaction and associated determinants of PCPs will help address major questions regarding the job satisfaction of PCPs who work in integrated care. However, to the best of our knowledge, the influence of integrated care on the job satisfaction of PCPs has yet to be the topic of any systematic review. To facilitate the integration of care from the primary care perspective and to support evidence-based healthcare decisions, consolidating the literature to systematically explore the correlation between the integration of care and the job satisfaction of PCPs is of crucial importance. This study aimed to address this gap by answering the following question based on Herzberg’s two-factor theory: how does the integration of care influence the job satisfaction of PCPs?

## Methods

### Research design

This study conducted a systematic review following the guidelines of the Preferred Reporting Items for Systematic Reviews and Meta-Analysis (PRISMA) statement [35]. Meta-analysis was not used for the review as selected studies were considered too heterogeneous in terms of methods and analysis and thus a more descriptive approach was suitable.

### Data source and searches

We searched the following databases: CNKI, WANFANG, PubMed, Web of Science, Cochrane Library, and Embase, among which CNKI and WANFANG were the commonly used databases for retrieving Chinese academic research. This study retrieved the data from inception to 19 March 2023. At the stage of identification, we used the following keywords to identify relevant studies: integrated care, primary care provider, and satisfaction. The search strategy is shown in Table 1.

**Table 1:** Search strategy

Databases	Search terms
Web of Science	#1((AB = (General Practitioner)) OR AB = (primary care provider)) OR AB = (community health workers) #2(AB = (integrated healthcare)) OR AB = (integrated care) #3AB = (satisfaction) #4 #1 and #2 and #3
PubMed	(((((integrated healthcare[Title/Abstract]) OR (integrated care[Title/Abstract])) AND ("general practitioner"[Title/Abstract] OR "primary care provider"[Title/Abstract] OR "community health workers"[Title/Abstract])))) AND (satisfaction)
Cochrane Library	#1 (integrated healthcare):ti, ab,kw OR (integrated care):ti, ab,kw #2(primary care provider):ti, ab,kw OR (General Practitioner):ti, ab,kw OR (community health workers):ti, ab,kw #3 (satisfaction) #1 and #2 and #3
Embase	#1 'integrated healthcare':kw,ab OR 'integrated care':kw,ab #2 'primary care provider':kw,ab OR 'general practitioners':kw,ab OR 'community health workers':kw,ab #3 'satisfaction':kw,ab #1 AND #2 AND #3
CNKI&WANFANG	(ti,ab,kw: Yilianti OR Yigongti^a^ OR integrated healthcare OR Urban medical group^b^ OR Cross-regional medical alliances^c^ OR Tele-medicine collaborative network^d^) AND (ti,ab,kw: primary healthcare workers OR healthcare workers) AND (ti,ab,kw: satisfaction)

Note:

^a^Countywide Yigongti was one type of Yilianti that referred to a medical partnership between delivering integrated care at the county level. Each Yigongti usually consisted of a county hospital, a township medical center, and a village clinic [81]

^b^Urban medical group was one type of Yilianti which was implemented in the form of the “1 + X” model. “1” referred to a top-tier hospital (e.g., a tertiary hospital), while “X” might include secondary hospitals, rehabilitation hospitals, nursing hospitals, and community hospitals [81, 82]

^c^Cross-regional specialty medical alliances were one type of Yilianti that referred to the horizontal integration of medical resources, especially the alliance of specialized medical facilities, within a certain region [83]

^d^Tele-medicine collaborative network was one type of Yilianti that referred to the vertical collaboration of medical facilities using the internet and other high-tech communication strategies [81]

### Study selection

We included studies following the PRISMA statement. First, we excluded duplicate studies from the database. In the screening stage, two researchers reviewed the title and abstract of the study and excluded the studies that were irrelevant to the topic. In the eligibility stage, we carefully reviewed the whole study and excluded studies that violated the inclusion criteria. Studies excluded at this stage are listed in Table 2 with detailed justifications. To be included in the review, the study had to meet the following inclusion criteria: (1) the research background of integrated care practice, including the integration of horizontal and vertical organizational management, governance mechanisms, financing and payment, and service provision; (2) the study population was the primary healthcare providers; and (3) focusing on the satisfaction of PCPs. Articles are excluded for several reasons: (1) changes in satisfaction of PCPs were not related to integrated care practice; (2) to study the evaluation of the cognition or effect of integrated care among PCPs, excluding the influence on satisfaction; and (3) the study of PCPs was not included.

**Table 2:** Excluded studies and justification for excluding

ID	Title	Justification for excluding
1	Facilitators and barriers for implementing the integrated behavioral health care model in the USA: an integrative review	The purpose of this study is to examine the promoting factors and obstacles in the implementation of integrated care, and it does not involve the study of the job satisfaction of PCPs
2	Evaluation of the geriatrician in the practice model of care for dementia assessment and management in rural Australia	The purpose of the study was to evaluate the effectiveness of the integrated care program and did not involve a study on the satisfaction of PCPs
3	Medical provider satisfaction with integrated care in a pediatric gastroenterology clinic	The research object of this study is pediatric gastroenterology providers, not PCPs
4	Effectiveness and cost-effectiveness of a proactive, goal-oriented, integrated care model in general practice for older people. A cluster randomized controlled trial: integrated systematic care for older people—the ISCOPE study	The purpose of this study was to explore the effectiveness and cost-effectiveness of the integrated care model from the perspective of the elderly, and it did not involve the study of job satisfaction of PCPs
5	Primary care provider perceptions of an integrated community pharmacy hypertension management program	The purpose of the study was to explore the perceptions of PCPs towards the integrated care plan, which did not involve a study of job satisfaction
6	General practitioners’ perspectives of the integrated health care system: a cross-sectional study in Wuhan, China	The purpose of this study was to explore the cognition and attitude of general practitioners towards the integrated care system, which did not involve the study of job satisfaction
7	Integrating Interdisciplinary Pain Management into Primary Care: Development and Implementation of a Novel Clinical Program	The purpose of the study was to develop and implement an integrated care system plan, and it did not involve a study on the job satisfaction of PCPs
8	Perceptions of collaboration between general practitioners and community pharmacists: findings from a qualitative study based on Spain	The purpose of the study was to explore the perceptions of GPs and pharmacists towards community pharmacist–GP collaboration, which did not involve a study on job satisfaction
9	Integrated primary care-behavioral health program development and implementation in a rural context	The purpose of the study was to explore the results of integrated primary care-behavioral health services to ensure the follow-up implementation of the program and did not involve the study of the job satisfaction of PCPs
10	An Examination of Perceptions in Integrated Care Practice	The purpose of this study was to explore the views of providers, support staff, and patients on integrated care, and PCPs were not clearly differentiated in the survey of providers
11	Child Health General Practice Hubs: a service evaluation	The purpose of the study was to evaluate the effectiveness of an integrated childcare system and did not involve measures of job satisfaction among PCPs
12	Examining the Impact of the Golden Compass Clinical Care Program for Older People with HIV: A Qualitative Study	The purpose of this study is to explore the impact of integrated care on the elderly with HIV, and it does not involve the study of the job satisfaction of PCPs
13	Interprofessional Teams: Lessons Learned From a Nurse-Led Clinic	The purpose of this study is to explore the experience and lessons learned in the implementation of integrated care and provide recommendations for the future, and does not involve the study of the job satisfaction of PCPs
14	Care Team Integration in Primary Care Improves 1-Year Clinical and Financial Outcomes in Diabetes: A Case for Value-Based Care	The content of the study focuses on the evaluation of clinical and financial outcomes of integrated care, rather than the study of job satisfaction of PCPs
15	Mental health and addictions capacity building for community health centres in Ontario	The purpose of this study is to evaluate the effect of the integrated care project, and it does not involve research on the job satisfaction of PCPs
16	Impact of Stressful Climates on Provider Perceptions of Integrated Behavioral Health Services in Pediatric Primary Care: An Exploratory Study	The purpose of this study was to investigate the impact of stress on PCPs providing integrated services, and it did not involve the study of job satisfaction
17	A Survey of Pharmacists in Integrated Care: Benefits, Barriers, and Facilitators of Integration	The study object is pharmacists, and it does not involve the study of the job satisfaction of PCPs
18	‘Trust and teamwork matter’: community health workers' experiences in integrated service delivery in India	The objective of this study is to explore the experience of community health workers in comprehensive services, and it does not involve the study of job satisfaction of PCPs
19	Care Coordinators in Integrated Care: Burnout Risk, Perceived Supports, and Job Satisfaction	The subjects of the study were care coordinators, which did not involve the study on the job satisfaction of PCPs
20	Research on medical staff satisfaction and policy cognition of County Yilianti in poverty-stricken area of Guangxi	The subjects of the study were medical staff in county-level hospitals and township health centers, excluding PCPs
21	Investigation and analysis of the current situation of medical staff working enthusiasm in basic public hospitals under the Yilianti model	The subjects of the study were medical staff of county-level public hospitals, not PCPs
22	Analysis on Current Situation of the Enthusiasm of Medical Staffs in a Yilianti, Beijing	To study the evaluation of the cognition or effect of integrated care among PCPs, excluding the influence on satisfaction
23	Evaluation of Yigongti Operation Effect from the Perspective of Township Medical and Health Institutions	To study the evaluation of the cognition or effect of integrated care among PCPs, excluding the influence on satisfaction
24	Investigation on the implementation effect of county medical community in Tongren City, Guizhou Province	To study the evaluation of the cognition or effect of integrated care among PCPs, excluding the influence on satisfaction
25	The current situation and effectiveness evaluation of medical staff's participation in Yilianti in Beijing	The purpose of this study is to explore the cognition of medical staff to the Yilianti policy, the status quo of work participation, and the evaluation of the effect and does not involve the study of job satisfaction
26	Investigation on the cognition and evaluation of medical community among medical staff in a division general hospital of Xinjiang production and construction corps	To study the evaluation of the cognition or effect of integrated care among PCPs, excluding the influence on satisfaction. In addition, study subjects did not include PCPs
27	Research on incentive mechanism of medical consortium based on incentive compatibility theory	The object of study, including county-level hospitals and the medical workers in towns and townships, is unable to make a clear distinction between PCPs from the research object
28	Problems and countermeasures in the construction of Zherong County General Hospital under the model of county medical community	To study the evaluation of the cognition or effect of integrated care among PCPs, excluding the influence on satisfaction. In addition, study subjects did not include PCPs
29	Qualitative Research on the Barriers to Primary Health Human Resources Construction and lts Countermeasures in The Context of Medical Alliance	The purpose of this study is to explore the problems and solutions of the construction of human resources for grassroots health under the background of the Yigongti, and it does not involve research on the job satisfaction of PCPs
30	Investigation and analysis of doctors' cognition of medical cluster indifferent medical and health institutions in Wuhan	The purpose of this study was to investigate and analyze the cognition of Yilianti among doctors at different levels of medical and health institutions in Wuhan, not involving the study of job satisfaction of PCPs
31	Survey on the cognition and operation satisfaction of medical alliance from the perspective of medical staff in member units of medical alliance	The purpose of this study is to explore the evaluation of medical staff on the operation mode and training system of the Yilianti, and does not involve the study on the job satisfaction of PCPs
32	Analysis of the Construction and Cognition of Medical Staffs in Anhui ProvinceC	The subjects of this study included medical staff in secondary hospitals, private hospitals, and community health service centers, and the PCPs could not be clearly distinguished from the subjects
33	Study on the Influence of the Linkage Mode of Medical Union on the Development of Primary Medical Institutions	The purpose of the study was to explore the cognition of medical staff to the Yilianti policy and the evaluation of the service ability of primary medical institutions, and did not involve the study of the job satisfaction of PCPs

### Quality assessment

Critical appraisal of the included studies was essential to a high-quality systematic review. Appraisal consisted of systematically examining studies to ensure they were trustworthy, valid, and reliable [36, 37]. Considering the varied types of the included studies (e.g., quantitative, qualitative, and mix-methods studies), we used the Mixed Methods Appraisal Tool (MMAT) version 2018 to evaluate the quality of the included studies [38]. MMAT was a quality assessment tool that could be used to evaluate five different types of studies: (a) qualitative, (b) randomized controlled, (c) non-randomized, (d) quantitative descriptive, and (e) mixed methods. Each criterion was rated as "yes", “no” or “unsure”. In this study, two researchers conducted MMAT independently. If there were any differences, they would discuss and resolve the differences. If the two evaluators could not resolve the differences, the third researcher would be invited to review the differences until a consensus was reached. Details of the quality evaluation of the included studies are shown in Table 3.

**Table 3:** Quality appraisal of the included studies

Included studies	Quantitative descriptive criteria				
	Is the sampling strategy relevant to address the research question?	Is the sample representative of the target population?	Are the measurements appropriate?	Is the risk of nonresponse bias low?	Is the statistical analysis appropriate to answer the research question?
Walter et al. (2019)	Yes	Yes	Yes	No	Yes
Sheehan et al. (2013)	Yes	Yes	Yes	No	Yes
Poot et al. (2016)	Yes	Yes	Yes	Unsure	Yes
Haag et al. (2021)	Yes	Yes	Yes	No	Yes
Farrar et al. (2001)	Yes	Yes	Yes	Yes	Yes
Selamu et al. (2019)	Yes	Yes	Yes	Yes	Yes
Waddimba et al. (2016)	Yes	Yes	Yes	Yes	Yes
Chen et al. (2021)	Unsure	Unsure	Unsure	Unsure	Unsure
Zhou et al. (2022)	No	Unsure	Yes	Yes	Yes
Wang et al. (2023)	Yes	Yes	Yes	Yes	Yes
Jia et al. (2014)	Yes	Yes	Unsure	Yes	Yes
Dong et al. (2022)	Yes	Unsure	Yes	Unsure	Yes
Wu et al. (2021)	Yes	Yes	Yes	Yes	Yes
He et al. (2021)	Yes	Yes	Yes	Yes	Yes

Qualitative criteria					
	Is the qualitative approach appropriate to answer the research question?	Are the qualitative data collection methods adequate to address the research question?	Are the findings adequately derived from the data?	Is the interpretation of results sufficiently substantiated by data?	Is there coherence between qualitative data sources, collection, analysis, and interpretation?
Mutemwa et al. (2013)	Yes	Yes	Yes	Yes	Yes
Vickers et al. (2013)	Yes	Yes	Yes	Yes	Yes
Rodríguez et al. (2018)	Yes	Yes	Yes	Yes	Yes

Mixed methods criteria					
	Is there an adequate rationale for using a mixed-methods design to address the research question?	Are the different components of the study effectively integrated to answer the research question?	Are the outputs of the integration of qualitative and quantitative components adequately interpreted?	Are divergences and inconsistencies between quantitative and qualitative results adequately addressed?	Do the different components of the study adhere to the quality criteria of each tradition of the methods involved?
Purcell et al. (2018)	Yes	Yes	Yes	Yes	Unsure
Kaitz et al. (2021)	Yes	Yes	Yes	Yes	No
Kool et al. (2008)	Yes	Yes	Yes	Yes	Yes
Jin et al. (2019)	Yes	Yes	Yes	Yes	Yes
Zhang (2022)	Yes	Yes	Yes	Yes	Yes
Zhou et al. (2018)	Yes	Yes	Yes	Yes	Yes
Zhang et al. (2022)	Yes	Yes	Yes	Yes	Yes
Wang (2017)	Yes	Yes	Yes	Yes	Yes
Ti (2019)	Yes	Yes	Yes	Yes	Yes
Ma et al. (2018)	No	Yes	Yes	Yes	No
Lin (2018)	Yes	Yes	Yes	Yes	Yes
Deng et al. (2021)	Yes	No	No	Yes	No

### Data synthesis

**Fig. 1:**
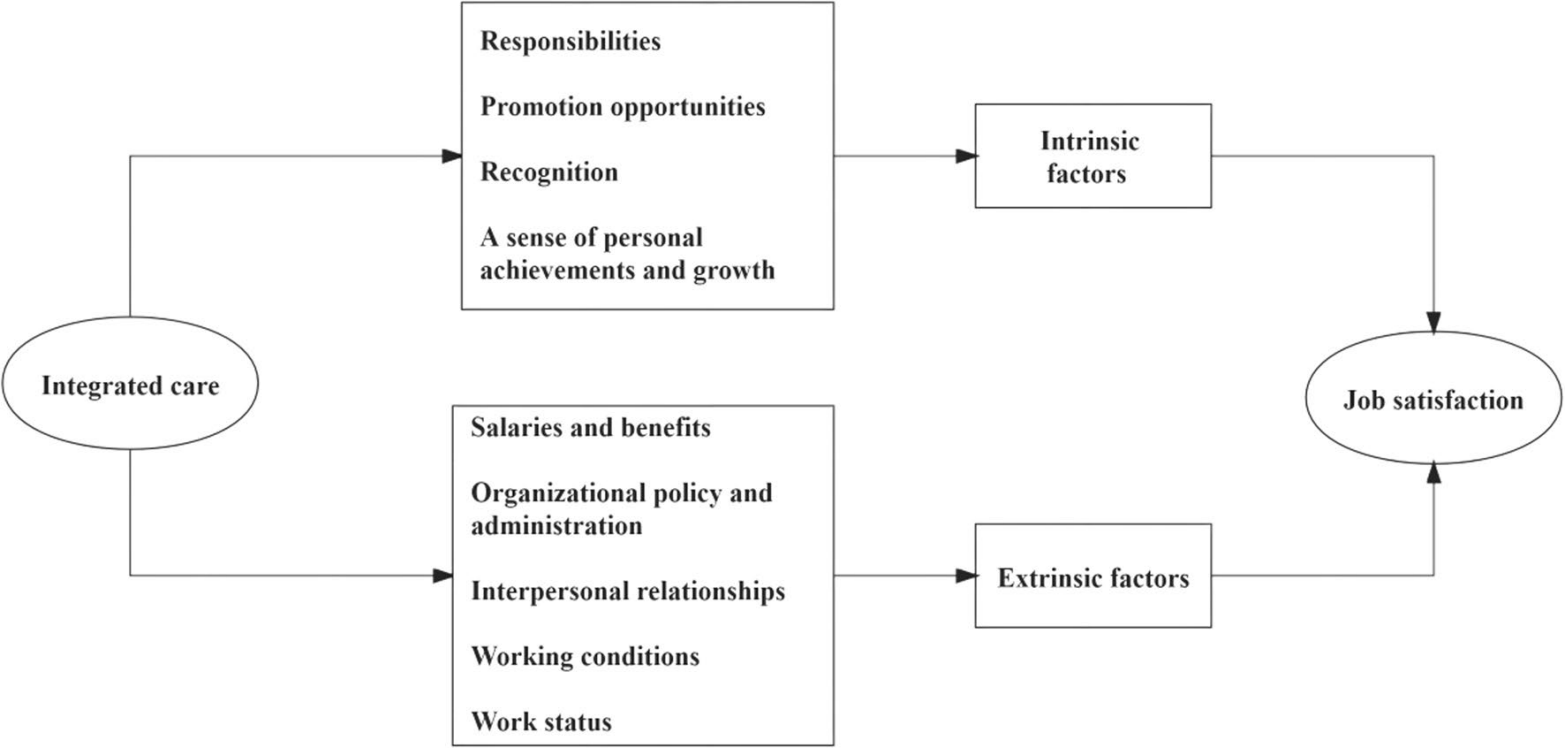
Analysis framework of influencing factors of PCP’s job satisfaction

This study used the integrative review method to extract and synthesize the data [39]. At the stage of data extraction, the key information extracted from each of the included studies were as follows: title, author(s), publication year, country(s), research method(s), integration types (horizontal/vertical), and measurement dimensions of satisfaction, which are summarized in Table 4. Horizontal integration refers to the integration between different types of healthcare services, such as medical services, social services, and other care, which is often in the form of multidisciplinary teams and/or care networks to take care of specific groups of patients. Vertical integration refers to the collaboration among primary, secondary, and tertiary medical institutions which aimed to increase the continuity of healthcare services [40, 41]. At the stage of data synthesis, we coded and divided the extracted information into categories and themes based on Herzberg’s two-factor theory. Two-factor theory is one of the most commonly used theoretical frameworks for exploring job satisfaction among health professionals [42, 43]. It hypothesizes two separate sets of mutually exclusive factors that either cause job satisfaction or dissatisfaction. Factors that primarily contributed to satisfaction alone called *motivation factors,* while the other factors that primarily contributed to dissatisfaction alone called *hygiene factors*. Herzberg also described *motivation factors* as intrinsic to the job and *hygiene factors* as extrinsic to the job [44]. Inspired by Herzberg’s analysis, we divided the dimensions of job satisfaction into intrinsic and extrinsic categories and used the notions of intrinsic and extrinsic factors to keep consistency. In this study, extrinsic factors were mainly related to working conditions, such as salaries and benefits, interpersonal relationships, and work environment. Intrinsic factors were mainly related to intrinsic work motivation, such as achievement and recognition, autonomy, and promotion opportunities. During the process of data synthesis, we found that some key information under certain themes needed to be specified. Therefore, we generated several sub-themes from coding. In the end, we formed a comprehensive picture of the analysis framework of influencing factors of PCP’s job satisfaction, as described in Fig. 1.

**Table 4:** Data extraction of the included articles

Study ID	Location	Periodyears	Integrationtype	Subjects	Measurement tool(s)	Measurement dimensions
*Quantitative studies*						
Farrar et al. (2001)	Canada	2001	horizontal	Family physicians	Self-designed questionnaire	The benefit to patients; disruption to office routines due to the presence of team members; the role of counselors and psychiatrists clinically and as educational resources; effects of the team on physicians' understanding of community resources, counseling techniques, diagnostic and treatment approaches
Jia et al. (2014)	China	2014	vertical	PCPs	Self-designed questionnaire	Current position; compensation and benefits; training and learning and personal growth; recognition and appreciation; working conditions; teamwork; organization and management
Poot et al. (2016)	Netherlands	2016	horizontal	GPs	Self-designed questionnaire	General satisfaction with General Practitioner care; Satisfaction about specific aspects of integrated care of GP care in General Practitioners: information exchange; coherence of care; multidisciplinary working
Waddimba et al. (2016)	USA	2016	vertical	Rural physicians and mid-level practitioners	Self-designed questionnaire	Self-reported satisfaction with Practice; needs(assess gratification of needs for autonomy and relatedness); work Meaningfulness; tolerance of uncertainty/ambiguity (the uncertainty of patient care); workload
Walter et al.(2019)	USA	2019	horizontal	Behavioral Health Integration Program(BHIP) Providers	Adapted questionnaires for self-efficacy and satisfaction [28]	Self-efficacy (Level of confidence in completing tasks); the comfort of teamwork; the effectiveness of teamwork; the medical treatment intention of patients and their family members increase access to quality BH services; get professional satisfaction
Selamu et al. (2019)	Ethiopia	2019	horizontal	Primary healthcare workers	Maslach Burnout Inventory (MBI); Job Satisfaction Questionnaire (JSQ)	Overall satisfaction: the application of personal expertise and interests and their suitability, as well as learning opportunities and job security Internal satisfaction: salary and benefits, promotion and development, reputation brought by work, etc External satisfaction: working environment and relationship with superiors and colleagues
Wu et al. (2021)	China	2021	vertical	PCPs	Self-designed questionnaire	Satisfaction with various work of medical consortium construction; satisfaction with the environment; satisfaction with treatment, performance distribution, and opportunities for further study
He et al. (2021)	China	2021	vertical	PCPs	Self-designed questionnaire	Leader/Colleague relationships; Doctor sinking from the superior hospital(helping effect of superior doctors); Working conditions and equipment configuration; Training opportunities; Training content; Ability improvement; Opportunity to use ability; Promotion opportunities; career development prospects; performance appraisal system; welfare benefits; Income level; Management status; Cultural atmosphere
Chen et al. (2021)	China	2021	vertical	County, township, and village medical personnel	Self-designed questionnaire	Working environment and conditions; management system of the medical community; personal career development; colleague relationship; social recognition
Haag et al. (2021)	USA	2021	horizontal	Physicians working in primary care clinics	Self-designed questionnaire	Workload and job demand; services efficiency and resources (services utilization, knowledge and education); meaning in work (patient care and professional development); social support and community at work (team support)
Sheehan et al. (2013)	Canada	2022	horizontal	Healthcare providers within primary care	Self-designed questionnaire	Expansion of knowledge and skills; reduction in professional isolation; addressing learning needs; recommendation of the session to others; overall satisfaction; learning and self-efficacy; performance
Dong et al. (2022)	China	2022	vertical	County-level and community-level medical personnel	Self-designed questionnaire	Working pressure; professional ability; salary package
Zhou et al. (2022)	China	2022	vertical	County, township, and community medical personnel	Self-designed questionnaire	Workload; skill level improvement, work pressure; professional level (service quality); work enthusiasm; working hours; career development prospects; doctor–patient relationship; changes in income; opportunities for further study and training; reasonable distribution of remuneration
Wang et al. (2023)	China	2023	vertical	Leading and member hospital medical staff	Self-designed questionnaire	Operating mechanism; medical level; the sense of achievement; working hours and workload; job pressure; income; internal performance incentive
*Qualitative study*						
Mutemwa et al. (2013)	Kenya	2013	vertical	Healthcare providers in hospitals, sub-district hospitals, and health centers	Interviews to explore provider experiences with integration to ascertain their significance to the performance of integrated health facilities	Summary of benefits and challenges of integration reported by providers
Vickers et al. (2013)	USA	2013	vertical	PCPs	Interviews to elicit opinions about mental health services before on-site system and resource changes occurred and repeated following changes that were intended to improve access to on-site mental health care	Opinions of mental health services after the resource and system changes: access to care domain and Referrals for care domain
Rodríguez et al. (2018)	USA	2018	vertical	Social workers, psychiatrists, and primary care providers	Interviews to learn about providers’ experiences to implement the program, and their recommendations for its sustainability	Providers described interdisciplinary integration arising from the program, with accompanying benefits and challenges
*Mixed-methods study*						
Kool et al. (2008)	Netherlands	2008	vertical	Employees working at the integrated emergency post	A specially developed survey based on a validated questionnaire; interview to explore staff’s idea of IEPs	Autonomy; clarity about their tasks staffing; patient care; use of personal capacities; social climate; information; culture work and organization
Wang (2017)	China	2017	vertical	County and township medical staff	Self-designed questionnaire; interviews to learn the achievements, problems, and countermeasures of such a policy	Career development; salary work pressure; the sense of achievement
Purcell et al. (2018)	USA	2018	vertical	PCPs, other primary care team members, and organizational stakeholders	Self-designed questionnaire; interview to elicit interviewees’ perspectives on the perceived effectiveness of the chronic pain care provided to their patients; job satisfaction, stress level, and burnout; confidence in and comfort and whether and how the implementation of Integrated Pain Team affected each of these	Perceived effectiveness of chronic pain care; providers confidence and skill in providing chronic pain care; satisfaction, stress, and burnout for providers and other team members
Zhou et al. (2018)	China	2018	vertical	Medical staff in district hospitals and community health management centers	Self-designed questionnaire; interviews to understand the changes in their organization and their work before and after the reform, their job satisfaction, and their opinions and suggestions for the reform	Work itself; interpersonal relationships; hospital development
Ma et al. (2018)	China	2018	vertical	The medical staff of community health centers	Self-designed questionnaire	Salary level; distribution system; working conditions; performance appraisal scheme; professional training; personal ability to play a platform; interpersonal relationship; overall satisfaction
Lin (2018)	China	2018	vertical	County and township medical personnel	Self-designed questionnaire; interviews to deeply understand the implementation of relevant measures in County Yigongti as well as their interest demands, cognition of the policy, work enthusiasm, and the confusion and problems existing in the actual operation	Personal income; personal working ability; working conditions; training opportunities; obtaining a higher degree
Jin et al. (2019)	China	2019	horizontal	PCPs	Self-designed questionnaire; interviews to better understand the expanded roles of PCPs and their influences on job satisfaction from the perspectives of administrators and service providers	Workload; income level; work autonomy
Ti (2019)	China	2019	vertical	Medical staff at county, township, and village levels	Self-designed questionnaire; interviews to learn the operation status, achievements, difficulties, and problems encountered in the reform, and improvement suggestions of the Yigongti	Income; benefits; distribution system; working environment; training opportunities; performance appraisal
Kaitz et al. (2021)	UK	2020	vertical	Psychologists and primary care physicians	Self-designed questionnaire; interview to explore providers' experiences working in their particular work setting and their collaboration with interdisciplinary providers	Level of administrative support; satisfaction with inter-professional collaboration; job satisfaction; provider holistic beliefs
Deng (2021)	China	2021	vertical	Medical staff in community health service centers	Self-designed questionnaire; interviews to learn the employees' satisfaction, opinions, and suggestions on the operation and management of the medical group	Working environment, management level; workload; salary level; career prospects; work status
Zhang et al. (2022)	China	2022	vertical	Rural doctor	Perceived organizational support Scale; interviews to better understand Village doctors' views on the "rural integration" reform, and their feelings on the changes in various aspects of township health centers after the implementation of the "rural integration" management	Organizational support; emotional support; organizational reward; procedural fairness; superior support
Zhang (2022)	China	2022	vertical	The medical staff of higher and primary medical institutions	Self-designed questionnaire; interviews to better understand the implementation and effectiveness of such policies, as well as the cognitive satisfaction of medical workers with such policies	Cognition and evaluation; willingness to work in the medical alliance; work experience

## Results

### Search results

**Fig. 2:**
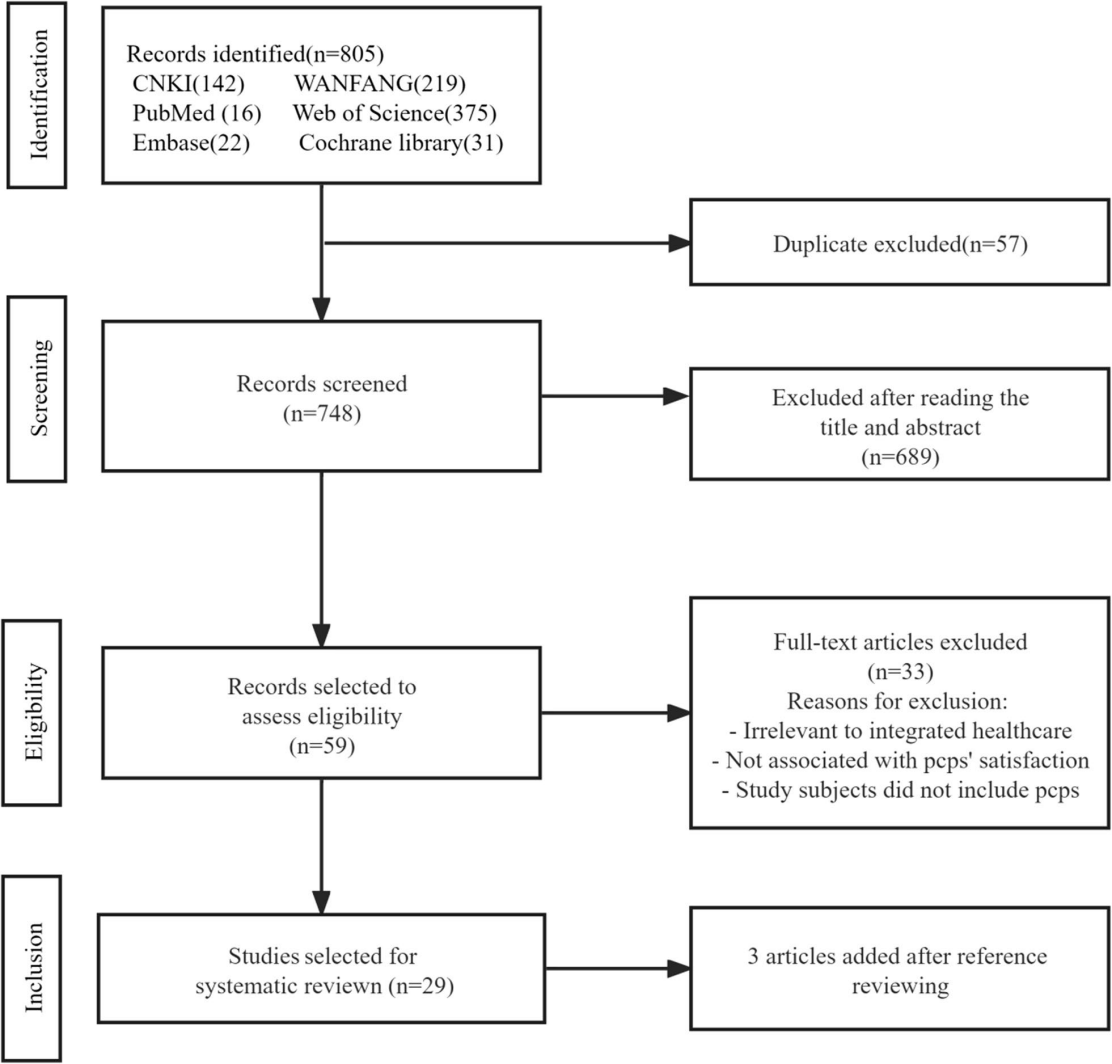
PRISMA flow diagram of study selection

The PRISMA flow chart in Fig. 2 illustrates the search process for this review. First, a total of 805 articles were retrieved through databases, among which 57 duplicate articles were removed. Second, after reviewing the title and abstract of the remaining 748 articles, we excluded 689 articles that did not match the inclusion criteria previously mentioned. Third, after the initial review, 59 full texts were examined carefully and 33 articles were excluded due to being irrelevant to integrated care, not associated with PCPs satisfaction, and PCPs were not study subjects. Fourth, 3 articles were added based on a reference review. In the end, we included 29 articles in this study.

### Description of the included studies

Of the 29 studies included in this review, there were 14 quantitative studies (4 longitudinal studies and 10 cross-sectional studies), 3 qualitative studies, and 12 mixed-method studies. The selected articles covered diverse countries including China, the United States, Canada, Ethiopia, Kenya, the Netherlands, and the United Kingdom. Concerning the integration type, there were 16 vertical integration studies and 13 horizontal integration studies. A summary of the above-described characteristics of the included studies is shown in Table 5.

**Table 5:** Characteristics of included studies

General characteristics	Descriptions	Number	Percentage
Study locations			
	China	16	55.17
	USA	6	20.69
	Canada	2	6.90
	UK	1	3.45
	Netherlands	2	6.90
	Ethiopia	1	3.45
	Kenya	1	3.45
Study design			
	Quantitative studies	3	10.34
	Qualitative studies	14	48.28
	Mixed-method studies	12	41.38
Integration type			
	Horizontal	13	44.83
	Vertical	16	55.17

Of the 16 vertical integration studies, 14 focused on the *Yilianti* of China, 1 focused on the urban medical groups in China, and 1 focused on the integration of care in a rural area of the USA. Of the 13 horizontal integration studies, 9 studies reported the integration of primary care and other kinds of healthcare, including public healthcare [9], mental healthcare [11, 45–48], pain care [12], and pharmaceutical care [10]. There were also 3 studies focusing on providing integrated care for vulnerable individuals, such as children [28, 49] and the elderly [50]. Besides the mode of “primary care + X”, there was also a study reporting the integration of HIV care and mental healthcare [30]. In addition, studies also explored the integration of care through the collaboration of healthcare professionals [51].

The included studies assessed the impact of integrated care on PCPs’ satisfaction through different methodologies and tools. Of the 14 quantitative studies, only 1 study used a scale named Job Satisfaction Questionnaire [52], and the other 13 studies used self-developed questionnaires to assess the satisfaction of PCPs. Of the 12 mix-method studies, semi-structured interview was the most used method for collecting qualitative data, while the quantitative data were mainly collected through self-developed questionnaires and only 1 study used the Perceived Organizational Support Scale [53].

### Effects of integrated care on the job satisfaction of PCPs

Due to different research aims, the effects of integrated care on the job satisfaction of PCPs were reported differently by the included studies in this review. We coded and divided the discussed effects into categories, themes, and sub-themes based on Herzberg’s two-factor theory, as presented in Table 6.

**Table 6:** Diverse influence of integrated care on the job satisfaction of PCPs

Categories	Themes	Sub-themes	Relevant studies
Intrinsic factors	Responsibilities		Waddimba et al. (2016); Jin et al. (2019); Zhou et al. (2018);
	Promotion opportunities		Zhou et al. (2022); Wang et al. (2023); He et al. (2021); Ma et al. (2018);
	Recognition	Patient feedback	Mutemwa et al. (2013); Vickers et al. (2013); Rodríguez et al. (2018)
		Service effect	Walter et al. (2019); Vickers et al. (2013); Rodríguez et al. (2018); Haag et al. (2021); Zhou et al. (2022); Wang et al. (2023); Purcell et al. (2018); Farrar et al. (2001);
	A sense of personal achievements and growth	Knowledge and skills improvement	Sheehan et al. (2013); Selamu et al. (2019); Zhou et al. (2022); Jia et al. (2014); Dong et al. (2022); Mutemwa et al. (2013); Vickers et al. (2013); Purcell et al. (2018); Zhou et al. (2018)
		Work achievement/meaning	Wang et al. (2023); Wang (2017); Waddimba et al. (2016)
		Learning and Training opportunities	Wu et al. (2021); Mutemwa et al. (2013); Rodríguez et al. (2018); Zhang et al. (2022); Ti (2019); Ma et al. (2018)
		Self-efficacy	Walter et al. (2019); Sheehan et al.(2022); Selamu et al. (2019); Purcell et al. (2018)
Extrinsic factors	Salaries and benefits	Income/Salary	Zhou et al. (2022); Jia et al. (2014); Dong et al. (2022); Mutemwa et al. (2013); Zhou et al.(2018); Wang (2017); Ti (2019); Ma et al. (2018); Wang et al.(2023); Lin (2018); Deng (2021); He et al. (2021); Chen et al. (2021); Zhang et al. (2022)
	Organizational policy and administration	Methods of payment distribution	Zhou et al. (2018); Ti (2019); Ma et al. (2018); Zhou et al. (2022)
		Management level	Deng et al. (2021); Wang et al. (2023)
		Performance appraisal system	Wang et al. (2023); Jia et al. (2014); Zhou et al. (2018); Ti (2019); Ma et al. (2018); He et al. (2021)
	Interpersonal relationships	Teamwork	Walter et al. (2019); Sheehan et al. (2013); Poot et al. (2016); Farrar et al. (2001); Vickers et al. (2013); Rodríguez et al. (2018); Kaitz et al. (2021); Zhang et al. (2022); Waddimba et al. (2016)
		Leader/Colleague relationship	He et al. (2021); Zhou et al. (2018); Ma et al. (2018); Chen et al. (2021)
	Working conditions		Chen et al. (2021); Jia et al.(2014); Wu et al.(2021); Mutemwa et al. (2013); Ti (2019); Deng (2021); Zhou et al.(2018); He et al.(2021); Ma et al. (2018)
	Work status	Workload	Haag et al. (2021); Waddimba et al. (2016); Mutemwa et al. (2013); Jin et al. (2019); Selamu et al. (2019)
		Work stress	Wang et al. (2023); Dong et al. (2022); Purcell et al. (2018); Wang (2017); Rodríguez et al. (2018); Deng (2021)
		Burnout	Haag et al. (2021); Selamu et al. (2019)

### Intrinsic job satisfaction

Intrinsic job satisfaction referred to the internal factors of a job that were relevant to job satisfaction which included 4 themes: responsibilities, opportunities for advancement, recognition, and a sense of personal achievements and growth.

#### Responsibilities

In this review, responsibilities are related to the work autonomy of PCPs. Studies reported that the integration of care affected the job satisfaction of PCPs by influencing their work responsibilities [29, 54, 55]. However, there was no consensus on whether the effects were positive or negative. For instance, a study focused on integrating multiple public healthcare, such as individual preventive services and population health interventions, into primary care claimed that the integration generated negative effects on PCPs’ work autonomy. Providing public healthcare imposed fixed work procedures and stringent requirements on PCPs, especially the increased paperwork and frequent evaluations generated by the bureaucratic structure of public health [9]. On the contrary, 2 studies reported that the job satisfaction of PCPs was increased along with their improved work autonomy due to the integration of care [13, 29].

#### Recognition

The theme “recognition” included 2 sub-themes: “patient feedback” and “service effect”. The recognition from patients or providers themselves was an important driving force behind the job satisfaction of healthcare providers [56, 57]. In this review, 5 studies showed that the integration of care significantly increased the accessibility of high-quality healthcare which benefited patients. The better health outcomes of patients increased the self-esteem and confidence of PCPs which positively influenced their job satisfaction [10, 28, 47–49]. 2 studies directly reported that integrated care increased the job recognition of PCPs, because they got more positive feedback from patients, especially from those with multi-morbidity [30, 48]. Besides the integration of care, a study showed that collaboration and cooperation among health professionals would increase the job recognition of PCPs by cultivating a teamwork culture [49]. Furthermore, about 77% of the surveyed PCPs expressed their recognition of integrated care and believed that a well-structured care team with a clear division of responsibilities would also be beneficial to the quality of primary care services [12].

#### A sense of personal achievements and growth

“Knowledge and skills improvement”, “work achievement/meaning”, “learning and training opportunities” and “self-efficacy” were classified into the theme “a sense of personal achievements and growth”. Studies found that PCPs held a higher self-expectation under integrated care, because the integration of care provided them with more opportunities to participate in teamwork and to get trained in a wide range of different clinical settings [12, 30, 49, 53, 58]. Besides the benefits of teamwork and training opportunities, the integration of care helped PCPs to gain more practical experiences from diagnosing and treating patients with complicated conditions [13, 30, 45, 48, 59, 60]. The increased knowledge and the improved skills could also help PCPs to build confidence to work towards their career goals and ultimately enhanced their job satisfaction [11, 12, 28, 45]. Despite those substantial benefits, the integration of care would also increase the perceived work value of PCPs and thereby enhanced their job satisfaction [29].

#### Promotion opportunities

Studies indicated that there was a close relationship between job satisfaction and the promotion opportunities of PCPs who worked in integrated care [32, 61, 62]. Studies showed that the current promotion evaluation system of PCPs over-emphasized their scientific research achievements. However, the majority of primary healthcare institutions (PHIs) were underdeveloped and lacked the necessary facilities and patient cases to support PCPs to conduct research. As a consequence, most of the PCPs complained about the hardship of getting promoted. The lack of promotion opportunities has brought down the job satisfaction of PCPs [34, 63].

### Extrinsic job satisfaction

Extrinsic job satisfaction referred to the external factors of a job that influenced job satisfaction. In this review, extrinsic job satisfaction was divided into 5 themes which included salaries and benefits, organization policy and administration, interpersonal relationships, working conditions, and work status.

#### Salaries and benefits

Some studies claimed that integrated care can affect the job satisfaction of PCPs who worked in integrated care through salaries and benefits levels [32, 60, 62]. 7 studies included in this review reported that PCPs had the lowest satisfaction with salaries and bonuses [13, 31, 32, 59, 61, 64, 65]. Studies also reported the imbalance between the increased workload and the relatively low income of PCPs who worked in integrated care, which also brought down the job satisfaction of PCPs [9, 30, 31, 33, 59, 61]. Furthermore, a study of Tianchang *Yigongti* specifically pointed out that 94.5% of PCPs were unsatisfied with *Yigongti* reform and the main reason for their dissatisfaction was related to low salaries [66].

#### Organizational policy and administration

Organizational policies mainly referred to the performance evaluation system of integrated care for PCPs in this review. Studies showed that the current performance evaluation system was unable to effectively reflect the work value of PCPs and, as a consequence, affected their job satisfaction [32, 34, 60, 61, 63]. A study of *Yilianti* in China reported that integrated care reform concentrated on integrating medical resources while paying less attention to improving institutional structures. As a consequence, *Yilianti* fell short of a fair payment distribution and promotion system which negatively influenced the job satisfaction of PCPs [63].

#### Interpersonal relationships

The theme “interpersonal relationships” included “relationships between colleagues”, “relationships with leaders”, and “teamwork”. In this review, 5 studies pointed out that integrated care prevented unfair competition by creating a friendly and supportive environment for teamwork [28, 45, 47–49]. Furthermore, a study compared the experience of PCPs who worked in integrated care and those who worked in standard care and found that PCPs who worked in integrated care were more satisfied with inter-professional collaborations [46]. In addition, 3 studies claimed that integrated care increased the mutual support between employees and employers which strengthened the professional identity and organizational belonging of PCPs and, as a consequence, improved their job satisfaction [13, 34, 65].

#### Working conditions

Studies reported that more positive perceptions of the environment and working conditions of medical staff brought higher job satisfaction [67, 68]. Concerning integrated care, studies also reported that it created a supportive working environment for PCPs [69, 70], thereby improving the work efficiency, service quality, and job satisfaction of PCPs. Similarly, studies that investigated the impact of *Yilianti* on the satisfaction of PCPs showed that vertical integration expanded the accessibility of high-quality medical resources, optimized the infrastructure and management level of PHIs, created a good working environment for PCPs, and improved their job satisfaction [13, 31, 32, 62–64, 71]. However, studies pointed out that many PCPs, especially those from less developed countries, complained about the organizational structure and poor facilities of PHIs [30, 34, 60]. Furthermore, a study that explored the experiences of providers who participated in the integration of HIV and reproductive health in Kenya pointed out that the infrastructure of health facilities urgently needed to improve [30].

#### Work status

In this review, the theme “work status” was associated with “workload”, “work stress”, and “burnout”. 12 of the included studies investigated the work stress of PCPs who worked in integrated care, but yielded inconsistent results. Among these studies, 3 studies reported that PCPs complained more often or easier to feel burnout when he or she experienced a heavier workload caused by the integration of care [9, 29, 33]. For instance, the underdeveloped integration of care would generate adverse effects on the continuity of care which turned out to be another source of work stress for PCPs [49]. Besides, 4 studies of *Yilianti* in China showed that the development of integrated care guided more patients to pay their first visits to PHIs which directly increased the workload of PCPs and burdened them with more work stress. However, the increasing workload of PCPs did not increase their income which negatively affected their job satisfaction [31, 33, 59, 63]. Whereas, two studies reported adverse results. They argued that integrated care (e.g., integrating mental healthcare into primary care) expanded the availability of healthcare providers with different backgrounds which released the work stress of PCPs and reduced their chances of burnout [10, 12].

## Discussion

This systematic review aimed to explore the influence of the integration of care on the job satisfaction of PCPs. To the best of our knowledge, this study is the first systematic review to explore the influence of integrated care on the job satisfaction of PCPs based on Herzberg’s two-factor theory. By synthesizing evidence from the included 29 studies, we summarized the measurement methods of job satisfaction and found that the integration of care affected different dimensions of the job satisfaction of PCPs. To help with clarification, we identified and classified the discussed effects into three levels (i.e., categories, themes, and sub-themes) based on Herzberg’s two-factor theory. Finally, we identified 2 categories (i.e., intrinsic and extrinsic), and 9 themes (i.e., responsibilities, promotion opportunities, recognition, a sense of personal achievements and growth, salaries and benefits, organizational policy and administration, interpersonal relationships, working conditions, and work status). Besides, we also identified 14 sub-themes to specify some key information.

Under the category of intrinsic job satisfaction, this review found both positive and negative effects of integrated care on PCPs’ satisfaction. Positive effects were mainly reflected by the theme of “recognition” and “personal accomplishment”, while negative effects were mainly reflected by the theme of “work autonomy” and “promotion opportunities”. The perception of work autonomy was identified as an important endogenous incentive factor for PCPs [72].

Studies in this review reported that PCPs from different PHIs which were in the process of integrated care reform demonstrated varied levels of work autonomy. This might be because PHIs were in the different stages of integrated care reform, such as the integration of services and the collaboration between different medical institutions were varied at different levels. Herzberg emphasized the importance of promotion opportunities as a motivating factor for employees [73]. However, this review found that compared with other countries, PCPs in China were less satisfied with promotion opportunities. This might be because of a higher threshold for promotion at the grass-roots level for PCPs. This review also suggested that increased levels of professional skills proved valuable to job satisfaction [30, 74]. According to Maslow’s hierarchy of needs theory, providing more career development opportunities could satisfy the self-actualization needs of PCPs, which is an effective way to improve the satisfaction of PCPs [75]. Another important aspect of intrinsic satisfaction was "recognition". This review found that self-affirmation of intrinsic value and recognition of extrinsic achievements could meet the needs of self-esteem, and thus had a positive impact on the job satisfaction of PCPs.

Under the category of extrinsic job satisfaction, studies included in this review also reported both negative and positive effects of integrated care on PCPs’ satisfaction. Negative effects were mainly reflected in “work status”, “work conditions”, “salaries and benefits”, and “organizational policy and administration”, while positive effects were mainly reflected in “interpersonal relationships”, “work status”, and “work conditions”.

Some studies in this review reported that integrated care increased the workload and work stress of PCPs [30, 31]. Whereas, some studies claimed that the integration of care helped to release the work stress of PCPs because of the improved cooperation and collaboration among medical institutions and health professionals [12, 76]. Those adverse effects of integrated care on the job satisfaction of PCPs might be due to the different types of integrating. Furthermore, studies in this review reported that “salaries and benefits” were a prominent factor that might generate job dissatisfaction. Due to the current unfair performance appraisal system, there existed an imbalance between income and workload which led to dissatisfaction and burnout of PCPs [9, 29]. This might be well-explained by the pay-return imbalance model: when more energy and effort was put into the work than the return, it could cause employees to have a sense of psychological imbalance, which could lead to negative emotions and dissatisfaction [77]. Moreover, studies in this review also demonstrated that working conditions were a commonly discussed extrinsic factor influencing job satisfaction. Previous studies of job satisfaction among PCPs explored the diverse influence of work conditions on PCPs and their work, such as the quality of medical services, occupation development, quality of life, and the turnover perception of PCPs [71, 78]. Taking the integration of care as a context, this review explored and collected specific findings about the working conditions of PCPs under the integrated care reform and the resulting job satisfaction. According to the studies included in this review, some countries did not pay enough attention to optimizing working conditions for PCPs [30, 34]. In addition, studies in this review also addressed the importance of mutual support among PCPs and emotional support from their supervisors to their job satisfaction under integrated care [79, 80]. Stable and secure emotional bonding would contribute to the working efficiency of PCPs [56].

### Limitations

This systematic review has several limitations. First, the studies included in this review vary considerably in the methods and tools used to measure the job satisfaction of PCPs. Therefore, it is difficult to standardize the results for satisfaction, limiting the comparability of the results. Second, most of the studies included in this review were cross-sectional studies, and differences in job satisfaction measurement tools may affect the generalizations of the findings. In addition, based on the results of the quality assessment of the articles, some of the studies included in this review did not fully meet the quality criteria and lacked information on sampling strategy, target group representation, and response rates. Finally, different populations, sample sizes, backgrounds, ethnic cultures, healthcare systems, types of integration, and institutions may have influenced the study results.

### Implications for future studies

Given that the included studies of this review were mainly cross-sectional studies with only 4 longitudinal studies, future studies on exploring the impacts of integrated care on PCPs were recommended to employ longitudinal study design. Given that the majority of the included studies used self-developed questionnaires to measure the impact of integrated care on the job satisfaction of PCPs with only 2 studies used valid scales, there was a need of developing a comprehensive and effective measurement tool to measure the effects of integrated care on the job satisfaction of PCPs. Given the important role of motivation in improving PCPs’ performance and achieving desired health system outcomes, future studies on integrated care should consider expanding their analysis to include the impact of changes in motivation on PCPs’ performance and other broader health system outcomes.

## Conclusion

This review synthesized the included 29 articles and explored the effects of the integration of care on the job satisfaction of PCPs. Based on Herzberg’s two-factor theory, this review identified 2 categories, 9 themes, and 14 sub-themes from the included studies. Findings suggested that the integration of care had both negative and positive effects on the job satisfaction of PCPs and the effects were different depending on the types of integration. Since PCPs played a vital role in the successful integration of care, their job satisfaction was an important issue that should be carefully considered when implementing the integration of care.

## Data Availability

The data sets generated and analyzed during the current study are available from the corresponding author upon reasonable request.

## Abbreviations

PCP: Primary healthcare providers
PHI: Primary healthcare institutions
BHIP: Behavioral Health Integration Program
BHCs: Behavioral health clinicians
IEP: Integrated emergency post
GP: General practitioner
MBI: Maslach Burnout Inventory
JSQ: Job Satisfaction Questionnaire
PRISMA: Preferred Reporting Items for Systematic Reviews and Meta-Analyses

## Footnotes

1 Yilianti was a typical model of integrated care in China which was de facto a kind of medical partnership emphasizing the coordination and cooperation between top-tier hospitals and grassroots medical facilities. There were four types of Yilianti in China: Countywide Yigongti, Urban medical groups, Cross-regional medical alliances, and Telemedicine collaboration networks [4].
